# Trans differentiating human adipose-derived mesenchymal stem cells into male germ-like cells utilizing Rabbit Sertoli cells: An experimental study

**DOI:** 10.18502/ijrm.v21i3.13197

**Published:** 2023-04-14

**Authors:** Alaa Mutee' Khudair, Mazen Medhat Alzaharna, Fadel Akram Sharif

**Affiliations:** Medical Laboratory Sciences Department, Faculty of Health Sciences, Islamic University of Gaza, Gaza City, Palestine.

**Keywords:** Adipose tissue-derived mesenchymal stem cell, Bone morphogenetic protein 4, Germ-line cells, Retinoic acid, Sertoli cells.

## Abstract

**Background:**

Mesenchymal stem cells (MSCs) are deemed as potential new therapeutic agents for infertility treatment and adipose tissue (AT) becomes a potential MSCs source. To direct MSCs through the differentiation process properly, an environment comparable to the in vivo niche might be indispensable.

**Objective:**

This study aims to differentiate human AT-derived MScs (hAD-MScs) into male germ-like cells in vitro using a combination of rabbit Sertoli cells conditioned medium (SCCM), bone morphogenetic protein 4, and retinoic acid.

**Materials and Methods:**

MScs were isolated from human ATs of fertile and infertile donors. The verified MScs were differentiated using a 2-step protocol; the first step included 20 ng/ml bone morphogenetic protein 4 treatment. The second step was performed utilizing 1 μM retinoic acid and/or SCCM. The morphological changes and the expression of germ cell (GC)-specific markers: octamer-binding transcription factor-4; stimulated by retinoic-acid-8*, *synaptonemal complex protein-3*, *andprotamine-1 were assessed in the treated cells using quantitative polymerase chain reaction.

**Results:**

Induction of hAD-MScs resulted in the upregulation of GC-specific genes where SCCM treatment showed the highest expression. The synaptonemal complex protein-3andprotamine-1 gene expression was detected after 19 and 26 days of induction, respectively. *PRM1* was detected in hAD-MScs cultured in SCCM earlier than in other treated groups. The treated cells became more elongated-like spindles and formed aggregates.

**Conclusion:**

hAD-MScs differentiated to GC lineage exhibited the ability to express GC-specific markers under in vitro conditions, and rabbit's Sertoli cells can be used for inducing transdifferentiation of hAD-MScs into germ-like cells.

## 1. Introduction

In the past few years, researchers have developed new options to treat infertility. One of the latest options is using stem cells to produce male germ cells (MGCs) through in vitro differentiation protocols (1, 2). Among different types of stem cells, mesenchymal stem cells (MSCs) are being increasingly developed as a new way to treat infertility cases, in particular, nonobstructive azoospermia (3). The selective differentiation of MSCs is largely dependent on the presence of growth and differentiation factors that mimic the environment of a particular cell lineage. As previously reported, using a culture system enriched with various factors with a preparation of neonatal testis for germ cell (GC) differentiation could actually mimic the in vivo developmental process in the case of MGCs (4). However, because human gonadal tissue is a limited resource, it is challenging to perform the differentiation protocol in in vitro primordial germ cells (PGC)-like cell generation utilizing neonatal human gonadal tissue. As a result, an alternative to human tissue was required to prepare the conditioned medium.

Some researchers have differentiated human stem cells using mice testicular tissue extract (1, 5). It was reported that even though the mouse is the most often used mammalian model for germline research, several features of human PGC development may differ from those of mice. In fact, certain mouse embryonic tissues involved in PGC formation have no obvious human equivalent (6). Therefore, it has shown that the other option is to employ gonadal somatic cells from other animals with flat-disc epiblasts and a molecular network comparable to the human PGC, such as pigs or rabbits (7). The rabbit is now the second animal, after the mouse, to have a regulatory network involved in germline specification expressed in a realistic time frame throughout early gastrulation and development stages. Rabbit embryology shares numerous similarities with that of humans, most notably in the general appearance of the peri-gastrulation embryo, which resembles a flat disc rather than the cup-shaped cylinder typical of rodents (8). In addition, it is broadly utilized in medical studies as a paradigm in human male reproductive system toxicology research (9, 10). So, in our study, we were concerned with differentiating human MSCs into GCs-like entities in vitro using the conditioned medium prepared from pre-pubertal rabbit testicular tissue that is similar to human tissue for along with exogenous factors that were reported to be the most important and key regulators of the in vivo differentiation processes such as bone morphogenetic protein 4 (BMP4) and retinoic acid (RA).

## 2. Material and Methods

### Isolation and preparation of Sertoli cells (SCs)-conditioned medium

Testes were collected from 42-days-old 5 healthy male prepubertal rabbits of the Dutch Rabbits breed (Oryctolagus Cuniculus) according to the American Veterinary Medical Association guidelines 2020. SCs were isolated using a 2-step enzymatic digestion protocol modified from published protocols (1, 11). Testes were then separated and washed 3 times with phosphate buffered saline (PBS) (Biological Industries, USA), they were then minced into small pieces and incubated in 10 ml of 1 mg/mL collagenase type IV (Worthington Biochemical, USA) solution at 37C for 60 min with shaking (120 cycle/min) then, centrifuged at 3000 rpm for 7 min. Following centrifugation, the supernatant was removed, and after 3 washes of the pellet in Dulbecco's modified Eagle's medium (DMEM), a second incubation was performed in trypsin-0.25% ethylene-diamine-tetra-acetic acid (EDTA) solution (Biological Industries, USA) at 37 C with gentle hand shaking every 2 min. After 10 min, pre-warmed complete DMEM (DMEM supplemented with 10% fetal bovine serum (FBS), 100 U/mL penicillin, and 100 mg/mL streptomycin) (Biological Industries, USA) was added to stop the trypsin activity, and the mixture was pipetted gently to shear the remaining cell clumps then centrifuged at 2200 rpm for 4 min. The supernatant containing Leydig cells was discarded and the pellet containing SCs was washed 3 times with PBS and filtered through a 75-μm sterile nylon mesh (Sigma-Aldrich, USA). The cell suspension obtained was cultured in complete DMEM at 37 C in a humidified incubator of 5% (v/v) CO_2_. Contaminating myoid cells in SCs culture were removed by treating it with 1M Glycine-2 mM EDTA solution followed by washing with complete DMEM. Remaining contaminating GCs were removed by changing the culture medium repeatedly for 7-10 days. Thereafter, every 3 days, the culture media was collected, centrifuged at 3000 rpm for 10 min, the supernatant was then transferred to a new conical tube, filtered using a 0.2-µm syringe filter, and stored at -20 C for later use. The supernatant was used as SCs conditioned medium (SCCM) in the in vitro differentiation experiments.

SCs morphology and culture purity were evaluated using Leishman cytological staining. Sterilized coverslips were placed in the wells of a 6-well culture plate and treated with FBS for at least 2 hr. After treatment, the cell suspension was added over each coverslip then incubated at 37 C in 5% CO_2_ for 24 hr to be attached. The culture medium was removed after 24 hr, the cells were gently washed thrice with PBS, and incubated with fixing solution at room temperature (RT) for 30 min, then air-dried. The next step was incubation of the coverslips in Leishman cytological staining for about 7 min then washed with water for 2 min. After dried, morphological characteristics were observed under a light microscope at high resolution.

### Isolation and culture of human adipose derived MSCs (hAD-MScs)

2 samples of adipose tissue (AT) were obtained from male fertile and infertile donors aged under 40 from abdominal surgery and liposuction samples, respectively. The AT samples were washed with sterile PBS supplemented with 100 U/mL penicillin, and 100 mg/mL streptomycin 3 times. Minced tissue was incubated in 1 mg/mL collagenase type I solution for 60 min at 37C with shaking at 120 cycle/min at orbital shaker. After incubation, complete DMEM was used to eliminate the activity of collagenase enzyme. The disaggregated tissue was filtrated through a 75-μm nylon mesh and centrifuged at 3000 rpm for 7 min, the pellet was resuspended in 1 ml of complete DMEM and incubated with 3 ml of erythrocyte lysis buffer (155 mM NH_4_Cl, 10 mM KHCO_3_, 0.1 mM EDTA) for 5 min at RT. After incubation, 10 ml of PBS was added to the cell suspension then centrifuged at 3000 rpm for 5 min, the pellet was then resuspended in 1 ml of complete DMEM. The cell suspension obtained was placed in culture dish and incubated in complete DMEM at 37 C in a humidified incubator of 5% CO_2_. The nonadherent cells were removed by changing the culture medium daily for at least 5 days, and then the medium was changed every 3^rd^ day.

### Characterization of adipose derived MSCs

According to the recommended criteria of the Mesenchymal and Tissue Stem Cell Committee, the MSCs express CD105, CD90, and CD73 markers on their surface and lack the expression of CD45 (a marker of hematopoietic stem cells and endothelial cell). For characterization, the expression of *CD105*, *CD90*, *CD73*, and *CD45* genes was evaluated using reverse transcriptase-polymerase chain reaction (RT-PCR) technique. Table I contains a list of the primers used in the experiment. To perform that, total RNA was extracted from a cell lysate using the ISOLATE II Biofluids RNA Phenol Free Kit (Bioline, USA) according to the manufacturer's instructions.The concentration of RNA was checked by a NanoDrop1000 spectrophotometer. Using the SensiFAST^TM^ cDNA Synthesis Kit (Bioline, USA), 1 µg of total RNA was reverse transcribed into cDNA in accordance with the manufacturer's instructions. Thermal cycling conditions were: Incubation at 25 C for 10 min, followed by 42 C for 15 min, 85 C for 5 min, and hold at 4 C. Samples were subjected to 40-cycle PCR reaction using RT-PCR GoTaqⓇ Green Master Mix (Promega, USA) according to the manufacturer's instructions. The PCR products along with a 100 bp DNA ladder were subjected to electrophoresis on 3% (w/v) agarose gel containing 10 mg/ml ethidium bromide, and the amplified fragments were visualized using a UV transilluminator documentation system and photographed.

### Evaluation of hAD-MScs viability after treatment with RA or BMP4

MSCs at the 4 ${}^{\text{th}}$ passage were cultured in 96-wells plate at density of 4500 cells/well in 100 µl of complete DMEM, and incubated at 37 C in 5% CO_2_ incubator for 24 hr to attach and recover. After 24 hr, cells incubated in DMEM were supplemented with different concentrations of either RA (10 ${}^{-4}$ , 10 ${}^{-5}$ , 10 ${}^{-6}$ , and 10 ${}^{-7}$ M) or BMP4 (5, 10, 25, 50, and 100 ng/mL) for 3 days (each concentration in duplicate) at 37 C and 5% CO_2_. After 3 days, the 3-(4,5-Dimethylthiazol-2-yl)-2,5-Diphenyltetrazolium Bromide (MTT) assay was carried out to evaluate the cell viability and to determine the optimum concentration of each factor that maintain the best cell viability to be used to induce MSCs differentiation. The wells were washed carefully with PBS then 100 μl of MTT was added to each well (final concentration 0.5 mg/mL) then incubated at 37 C and 5% CO_2_ for 3 hr. After incubation, MTT was removed and 100 μl of dimethyl sulfoxide (DMSO) was added to solubilize the formazan blue crystals that had been produced. The plate was agitated on an orbital shaker for 30 sec, and then incubated for 15 min at RT. The absorbance was measured using enzyme linked immunosorbent assay reader at 570 nm. Then the number of live cells per well was calculated as a percentage of the control. To determine the optimal concentration to employ while differentiating MSCs into germ-like cells, a dose-response curve was created (1).

### In vitro differentiation experiment

A 2-step protocol modified from (12) was used to stimulate the differentiation of hAD-MScs to produce male germ-line cells. MSCs at passages 4 were divided into 3 main treatment groups with a control group: RA treated group, RA/SCCM treated group, SCCM treated group, and control group, cultured at a concentration of 15 × 10_3_ cells/well in a 24-well plate in complete DMEM at 37C with 5% CO_2_ for 24 hr to adhere.

In the first step of differentiation protocol, the cells were treated for 5 days with a concentration of 20 ng/ml BMP4 that was obtained by MTT test. 2 distinct treatment media were prepared, one contained 20 ng/ml of BMP4 and another without BMP4. RA treated group along with RA/SCCM treated group were incubated in complete DMEM containing 20 ng/ml BMP4 for 5 days. While both the SCCM treated group and control group were incubated in DMEM without the addition of BMP4.

In the second step, a concentration of 10 ${}^{-6}$ M RA was used. RA treated MSCs were incubated in complete DMEM supplemented with 10 ${}^{-6}$ M RA only, RA/SCCM treated MSCs were incubated in a medium containing 50% SCCM, 50% complete DMEM supplemented with 10 ${}^{-6}$ M RA, and the SCCM treated MSCs were incubated in a medium containing 50% SCCM, 50% complete DMEM. While the control group of MSCs were incubated in complete DMEM without BMP4 and/or RA and/or SCCM. The culture medium was changed every 3rd day. All the MSCs groups were incubated for a treatment period of 14 days and 21 days (total treatment time was 19 and 26 days, respectively).

### Gene expression analysis by quantitative RT-PCR

After each period of differentiation treatment into MGCs (19, and 26 days), the cells were trypsinized, and total RNA was isolated from the 4 groups using ISOLATE II Biofluids RNA Phenol Free Kit, its concentration and quality were checked, and 1 μg of total RNA was reverse transcribed to cDNA using SensiFAST^TM^ cDNA Synthesis Kit. Samples were subjected to quantitative polymerase chain reaction using PCR Go TaqⓇ Green Master Mix (Promega, USA) for 40 cycles using the following PCR conditions: 2 min at 95 C followed by 95 C for 5 sec, 59-60 C for 20 sec, and 72 C for 20 sec. Table I contains a list of the primers used in the experiment. After the reaction was completed, the amplified DNA along with a 100 bp DNA ladder were loaded on 3% agarose gel stained with 3 µL of 10 mg/ml ethidium bromide. The gel was run at 70 V for 30 min at RT and resulting DNA bands sizes were viewed under UV transilluminator documentation system and photographed. The photos of the gel bands were analyzed using image J software for quantitative analysis.

**Table 1:** Intended genes, primers sequences, and sizes of PCR amplification products


**Name**	**Intended gene**	**Sequence of Primer 5' → 3'**	**Tm ( C)**	**Product size (bp)**
		63	
**CD105**	*ENG*	Forward: CCACTAGCCAGGTCTCGAAG
			Reverse: GATGCAGGAAGACACTGCTG	60	192
		57			
**CD90**	*THY1*	Forward: ATGAACCTGGCCATCAGCA
			Reverse: GTGTGCTCAGGCACCCC	60	218
		63			
**CD73**	*NT5E*	Forward: CAGTACCAGGGCACTATCTGG
			Reverse: AGTGGCCCCTTTGCTTTAAT	56	194
		58			
**CD45**	*PTPRC*	Forward: CTGACATCATCACCTAGCAG
			Reverse: TGCTGTAGTCAATCCAGTGG	58	257
		60			
**OCT4** * *	*OCT4*	Forward: CCCGAAAGAGAAAGCGAACC
			Reverse: GCAGCCTCAAAATCCTCTCG	60	217
		60			
**STRA8** * *	*STRA8*	Forward: CGCTCTTCAACAACCTCAGG
			Reverse: ATGCCCATCTTCCAGGTTGA	58	161
		58			
**SCP3** * *	*SCP3*	Forward: TGCTGGAAGGAGTTGGAGTT
			Reverse: CCCACTGCTGAAACAAAGTCA	59	192
		60			
**PRM1** * *	*PRM1*	Forward: CAGAGTTCCACCTGCTCACA
			Reverse: ACTTCTTTGTCTCTGGCGGT	58	150
bp: Base pair, *ENG*: Endoglin, *NT5E:* Ecto-5 ' -nucleotidase, *OCT-4*: Octamer-binding transcription factor 4, *PRM1*: Protamine 1, *PTPRC:* Protein tyrosine phosphatase, receptor type C, *OCT4*: Octamer-binding transcription factor 4, *SCP3*: Synaptonemal complex protein 3, *STRA8*: Stimulated by Retinoic Acid 8, *THY1:* Thymocyte differentiation antigen 1, Tm: Melting temperature					

### Ethical considerations

The study received the approval of the Ethical Research Committee at the Islamic University of Gaza and the Helsinki committee in the Gaza Strip (number: PHRC/HC/736/20). Informed written consent of the AT donors was also secured. The animal procedures were performed according to the American Veterinary Medical Association guidelines 2020.

### Statistical analysis

For quantitative polymerase chain reaction analysis, Image J software (https://imagej.nih.gov/ij/) was used. The Statistical Package for Social Sciences (SPSS Inc., US) 22.0 software was used to analyze the results and different statistical analysis were performed. The independent samples *t* test and one-way repeated measures analysis of variance were used to determine the statistically significant differences between variables. P values ≤ 0.05 were considered statistically significant.

## 3. Results 

**Figure 1:**
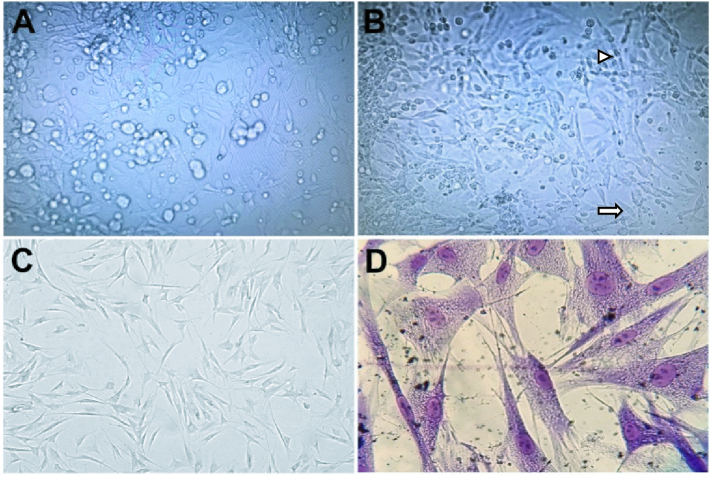
Isolated rabbit Sertoli cells (SCs) in the culture. A) Cell attachment of the extracted SCs at 24 hr, (Mag: × 200), B) Day 2 culture, SCs begin to attach to the bottom of the culture flask and 2 distinct cell phenotypes were detected after 24 hr, the arrow indicates a SC, and the arrow head indicates a GC, (Mag: × 100), C) SCs at Passage 2, the cells made a monolayer entity at the bottom of the culture flask and showed irregular shapes with predominately fibroblastic morphology, (Mag: × 200), D) SCs stained with Lishman stain (40X). The SCs had irregular polygonal shapes, well-formed cytoplasmic extensions with a granular appearance in the cytoplasm, large nuclei, and 3 nucleoli, they produced extensions, they were flattened and attempted to make contact with other cells, (Mag: × 400).

A few hours after enzymatic digestion of rabbit testis and culturing of cell suspension in the culture flask, SCs begin to attach to the bottom of the culture flask; 2 distinct cell phenotypes were detected after 24 hr, round GCs and fibroblast-like somatic SCs adhered to the bottom of the culture flask (Figure 1A and 1B). On day 2, SCs began to flatten, spread out, and had a fibroblastic appearance. They proliferated immensely within 4-7 days and formed a monolayer. The GCs were removed by exchanging of culture medium every day. After passage, the cells made monolayer entity at the bottom of culture flask and showed irregular shapes with predominately fibroblastic morphology (Figure 1C).

The morphological characteristics of cultured SCs were examined under inverted and light microscope after staining with Leishman stain. In general, the SCs had irregular polygonal shapes, well-formed cytoplasmic extensions with a granular appearance in the cytoplasm, large nuclei, and 3 nucleoli, they made elongations, flattened, and attempted to create contact with the other cells (Figure 1D).

**Figure 2:**
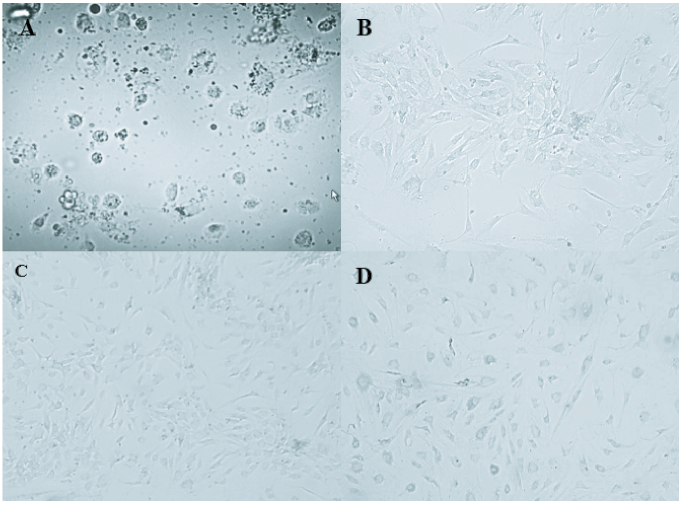
Isolated human adipose-derived mesenchymal stem cells (hAD-MScs) in the culture. A) Attachment of the hAD-MScs to the bottom of the culture flask, hAD-MScs exhibit the ability to adhere to the bottom of the plastic culture dish, they were initially round and begin to adhere to the bottom of the culture flask and some spindle-shaped cells appeared among the mononuclear cells on day 2, (Mag: × 200), B) Flattened hAD-MScs at day 4, cells began to flatten, spread out and take a fibroblastic appearance, hAD-MSCs were seen as spindle-shaped cells under inverted microscope and the morphology did not change during cell passages, (Mag: × 100),C) hAD-MScs proliferation within 5-7days, they started to proliferate within 5-7 days and formed a monolayer with a uniform fibroblast-like morphology, (Mag: × 40), D) hAD-MScs at passage 3 (4X), the cells made a monolayer at the bottom of the culture flask and showed a fibroblastic morphology (Mag: × 100), scale bar: 1000 µm.

MSCs were isolated from human AT. After one day of culturing, MSCs exhibited the capacity to adhere to the plastic culture flask's bottom, they were initially round and begin to adhere to the bottom of the culture flask (Figure 2A) and some spindle-shaped cells appeared among the mononuclear cells on day 2. On day 4, cells began to flatten, spread out, and take a fibroblastic appearance, and the morphology did not change during cell passages (Figure 2B). They started to proliferate within 5-7 days and formed a monolayer with a uniform fibroblast-like morphology (Figure 2C) after staining with Leishman stain. The SCs appeared to have irregular polygonal shapes, well-formed cytoplasmic extensions with a granular appearance in the cytoplasm, large nuclei, and 3 nucleoli, they produced extensions, they were flattened and attempted to make contact with other cells (Figure 2D).

### hAD-MScs surface markers evaluation

**Figure 3:**
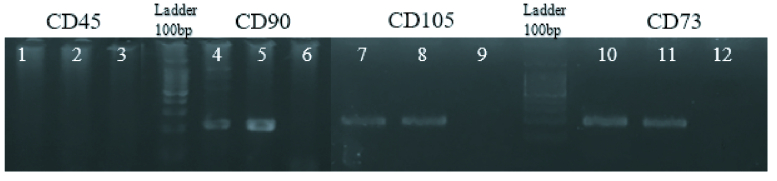
PCR products of isolated human adipose-derived mesenchymal stem cells (hAD-MScs) surface markers (CD45, CD90, CD105, and CD73) on ethidium bromide-stained 3% agarose gel. Lanes 1 & 2: CD45 (211 bp), Lane 3: Negative control of CD45, lanes 4 & 5: CD90 (218 bp), lane 6: Negative control of CD90, lanes 7 & 8: CD105 (192 bp), lane 9: Negative control of CD105, lanes 10 & 11: CD73 (194 bp), Lane 12: Negative control of CD73. CD 45 is a marker of hematopoietic stem cells and endothelial cells not mesenchymal stem cells according to the recommended criteria of the mesenchymal and tissue stem cell Committee.

The results indicated that hAD-MScs expressed the MSCs markers *CD73*, *CD105*, and *CD90*, but *CD45*, the hematopoietic stem cell marker, was not expressed (Figure 3). All the surface marker genes expression assessed in the isolated AD-MSCs conformed to the criteria of MSCs established earlier.

### Effect of various doses of RA on the hAD-MScs viability

**Figure 4:**
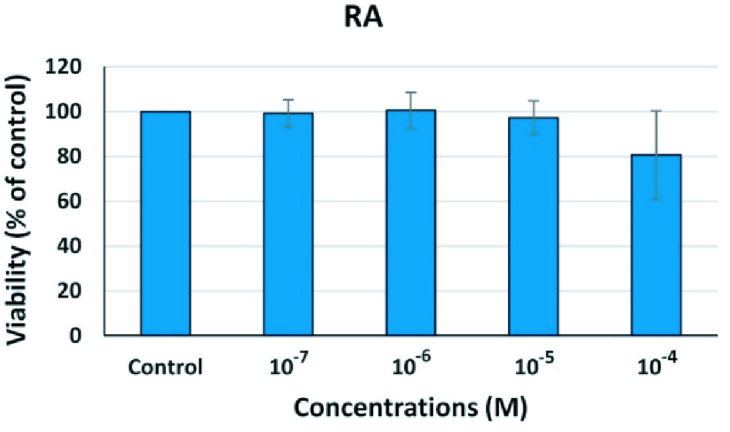
Effect of various doses of retinoic acid (RA) on human adipose-derived mesenchymal stem cells (hAD-MScs) viability. The viability of the cells was determined using MTT assay and the concentration of RA that had the least effects on the cell's viability was selected. The percentage of cell viability was significantly decreased at 10 ${}^{-4}$ M RA compared with dosages of 10 ${}^{-5}$ , 10 ${}^{-6}$ , and 10 ${}^{-7}$ M RA. However, percentage of cell viability was not significantly different between dosages of 10 ${}^{-5}$ , 10 ${}^{-6}$ , and 10 ${}^{-7}$ M RA.

The MTT test findings revealed that at 10 ${}^{-4}$ M RA, compared to doses of 10 ${}^{-5}$ , 10 ${}^{-6}$ , and 10 ${}^{-7}$ M RA, the percentage of cell viability was considerably lower (p = 0.04). However, there was no considerable difference in the cell viability percentage between doses of 10 ${}^{-5}$ , 10 ${}^{-6}$ , and 10 ${}^{-7}$ M RA (p = 0.07). For an induction of differentiation of MSCs into germ-like cells 10 ${}^{-6}$ M RA was selected (Figure 4).

### Effect of various doses of BMP4 on the hAD-MScs viability

**Figure 5:**
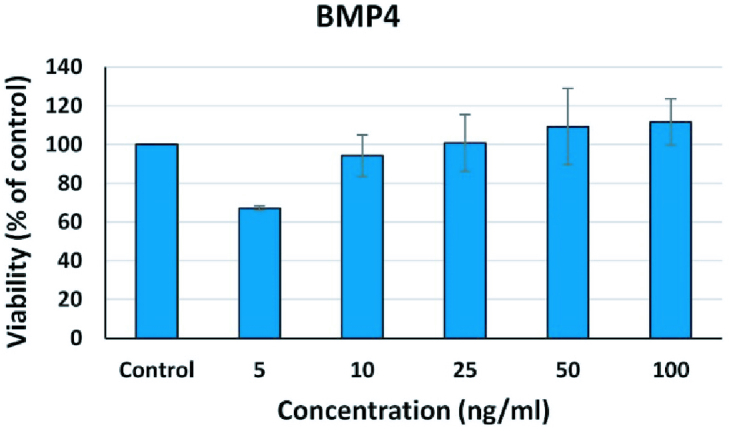
Effect of various doses of bone morphogenetic protein 4 (BMP4) on human adipose-derived mesenchymal stem cells (hAD-MScs) viability. The viability of the cells was determined using MTT assay and the concentration of BMP4 that had the least effects on the cell's viability was selected. The percentage of cell viability was significantly decreased in dosage of 5 ng/ml BMP4 compared with dosages of 10, 25, 50, and 100 ng/ml BMP4 and control groups. In addition, there is a significant increase in viability in 50 and 100 ng/ml compared to 10 and 25 ng/ml BMP4 and control groups. However, percentage of cell viability was not significantly different between dosages of 10, 25 ng/ml BMP4 and the control group.

The MTT test findings revealed at dosage 5 ng/ml BMP4 compared with dosages of 10, 25, 50, and 100 ng/ml BMP4 and control groups, the percentage of cell viability was considerably lower (p < 0.01). In addition, a significant increase was observed in viability in 50 and 100 ng/ml compared to 10 and 25 ng/ml BMP4 and control groups (p < 0.01). However, no considerable difference was observed in the cell viability percentage between doses of 10, 25 ng/ml BMP4 and the control group (p = 0.36). A value between 10 and 25 ng/ml BMP4 dosages, 20 ng/ml was selected for induction of differentiation of MSCs into germ-like cells (Figure 5).

### Evaluation of differentiation into GCs

#### Cell morphology evaluation in the treatment groups

**Figure 6:**
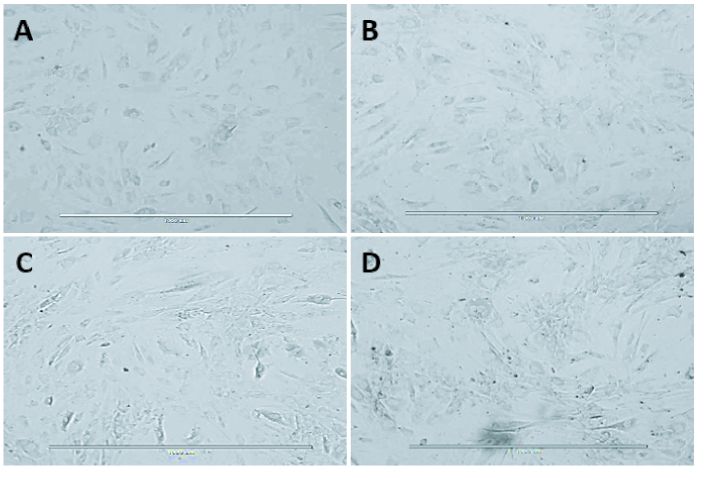
Morphology of human adipose-derived mesenchymal stem cells (hAD-MScs) after 5 days of treatment with 20 ng/ml BMP4. A) hAD-MScs from healthy fertile donor before BMP4 treatment, (Mag: × 200), B) hAD-MScs from infertile patient before BMP4 treatment, (Mag: × 200), C) hAD-MScs from healthy fertile donor after 5 days of 20 ng/ml BMP4 treatment, some cells were more oval unlike fibroblastic cells and formed cell aggregates, (Mag: × 200), D) hAD-MScs from infertile patient after 5 days of 20 ng/ml BMP4 treatment, some cells were more oval unlike fibroblastic cells and cell aggregates were formed, (Mag: × 200), scale bar: 1000 µm.

The change in cell morphology was evaluated every 3 days using an inverted phase contrast microscope. Figure 6 represents morphological characteristics of the hAD-MScs before treatment with 20 ng/ml BMP4 (Figures 6A, B) and after the cells were cultured in a dosage of 20 ng/ml BMP4 (Figures 6C, D), these cells were compared with the control group morphologically after 5 days of culture. The morphological change was noticeable after culturing in BMP4, such as some cells were more oval unlike fibroblastic cells and formed cell aggregates (Figures 6C, D).

**Figure 7:**
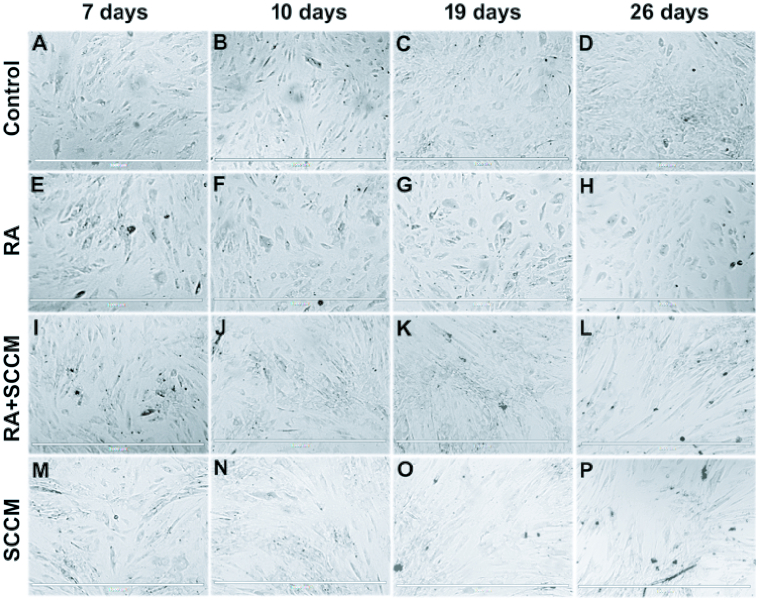
Morphological changes of human adipose-derived mesenchymal stem cells (hAD-MScs) of fertile donor after in vitro treatment by different methods on days 7, 10, 19, and 26. A-D) hAD-MScs incubated in DMEM medium containing 10% FBS. E-H) hAD-MScs were treated with bone morphogenetic protein 4 (BMP4) then retinoic acid (RA). The morphology of hAD-MScs gradually altered from fibroblast-like to be more oval with clear cellular boundaries and have a tadpole-like shape after 7 days, which in the following induction times did not alter considerably. I-L) hAD-MScs were treated with bone morphogenetic protein 4 (BMP4) then retinoic acid (RA) in combination with Sertoli cell conditioned medium (SCCM). Cells fibroblastic shape changed to become like slender spindles and a few cells that resembled tadpoles were present within 10-12 days, which transformed to slender spindles in subsequent times. M-P) hAD-MScs were treated with only Sertoli cell conditioned medium (SCCM). Virtually most of the cells lengthened and took the shape of spindles within 7 days, the cells appeared to be more elongated than in RA/SCCM group. (Mag: × 200), scale bar: 1000 µm.

**Figure 8:**
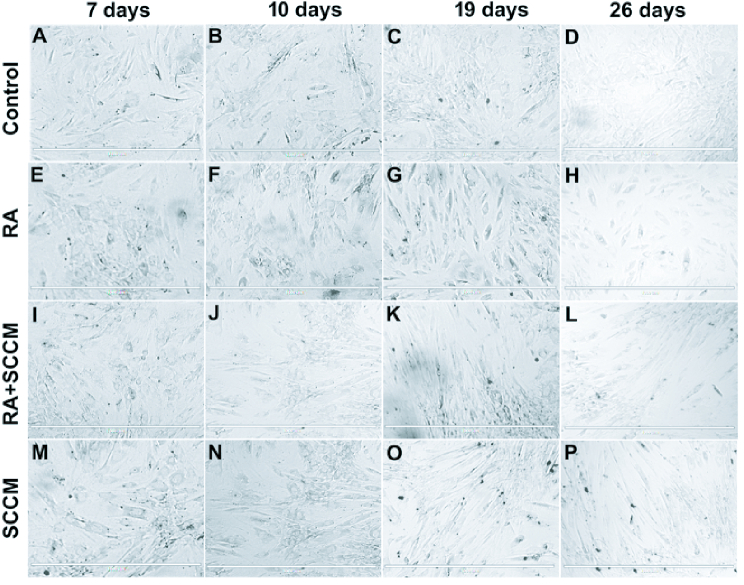
Morphological changes of human adipose-derived mesenchymal stem cells (hAD-MScs) of infertile patient after in vitro treatment by different methods on days 7, 10, 19, and 26. A-D) hAD-MScs incubated in DMEM medium containing 10% FBS. E-H) hAD-MScs were treated with bone morphogenetic protein 4 (BMP4) then retinoic acid (RA). The morphology of hAD-MScs gradually altered from fibroblast-like to be more oval with clear cellular boundaries and have a tadpole-like shape after 7 days, which in the following induction times did not alter considerably. I-L) hAD-MScs were treated with bone morphogenetic protein 4 (BMP4) then retinoic acid (RA) in combination with Sertoli cell conditioned medium (SCCM). Cells fibroblastic shape changed to become like slender spindles and a few cells that resembled tadpoles were present within 10-12 days, which transformed to slender spindles in subsequent times. M-P) hAD-MScs were treated with only Sertoli cell conditioned medium (SCCM). Virtually most of the cells lengthened and took the shape of spindles within 7 days, the cells appeared to be more elongated than in RA/SCCM group. (Mag: × 200), scale bar: 1000 µm.

Figures 7 and 8 represents the morphological changes of hAD-MScs of fertile donor and infertile patient respectively after the second step of the in vitro treatment by different methods on days 7, 10, 19, and 26. Figures 7 and 8 (A-D) represent the proliferation of untreated hAD-MScs on days 7, 10, 19, and 26 respectively. While in the treated groups, the cell proliferation in the RA treated group was lower than the other treatment groups and untreated group after 7 days. In addition, the morphology of hAD-MScs gradually altered from fibroblast-like to be more oval with clear cellular boundaries and have a tadpole-like shape after 7 days (Figures 7E and 8E), which in the following induction times did not alter considerably (Figures 7 F-H and 8 (F-H)).

hAD-MScs treated with RA/SCCM showed higher proliferation, and most of the cells retained their fibroblastic appearance at day 7 (Figures 7I and 8I). Their fibroblastic shape changed to become like slender spindles, and a few cells that resembled tadpoles were present within 10-12 days (Figures 7J and 8J), which transformed into slender spindles in subsequent times (Figures 7 and 8 (K-L)).

In the SCCM treated group, virtually most of the cells lengthened and took the shape of spindles within 7 days, the cells appeared to be more elongated than in RA/SCCM group, and the presence of aggregates increased in subsequent culture times (Figures 7 and 8 (M-P)).

#### Expression of GC specific genes in treated hAD-MScs

**Figure 9:**
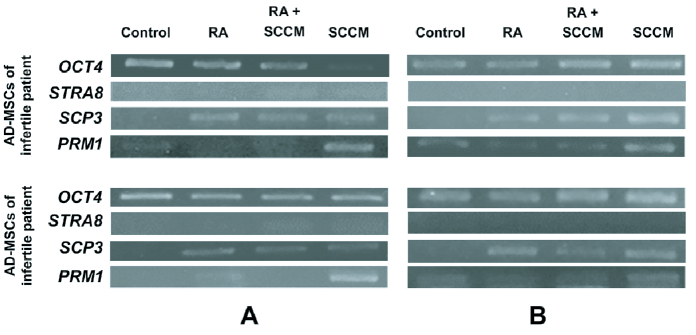
Photos representing PCR amplicons. The expression of germ cells specific genes in human adipose-derived mesenchymal stem cells (hAD-MScs) of healthy fertile donor after treatment with RA (hAD-MScs treated with 20 ng/ml BMP4 then treated with 10 ${}^{-6}$ M RA), RA/SCCM (hAD-MScs treated with 20 ng/ml BMP4 then treated with a combination of 10 ${}^{-6}$ M RA and SCCM); and SCCM (hAD-MScs treated with SCCM alone), compared to control without treatment. A) Expression of the genes after 19 days of treatment, B) Expression of the genes after 26 days of treatment.

The expression levels of GCs specific markers, *OCT4*, *STRA8*, *SCP3*, and *PRM1* were measured at 19 and 26 days after culture to check for the existence of differentiated cells in the cultured cells in the treatment and control groups. Our results indicated that all treated cells were positive for *OCT4* and *SCP3*, while *PRM1* was positive only in SCCM treated group after 19 days of treatment (Figure 9A). After 26 days of treatment, all treated hAD-MScs were positive for all the tested markers except *STRA8* compared to untreated hAD-MScs (Figure 9B).

**Figure 10:**
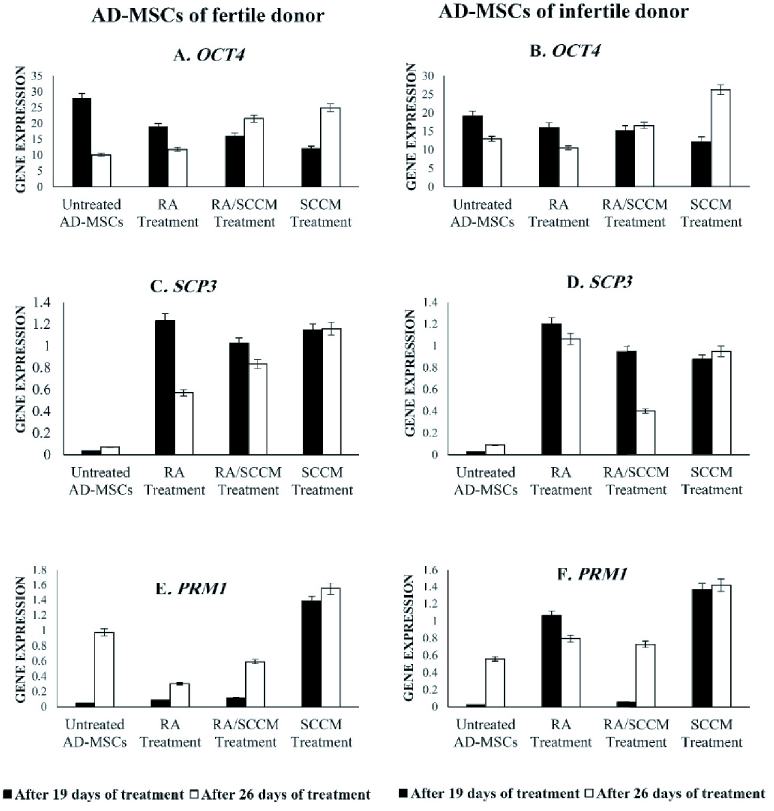
Quantitative-PCR results of germ cells markers (*OCT4*, *SCP3*, and *PRM1*) genes in treated human adipose-derived mesenchymal stem cells (hAD-MScs) derived from healthy fertile and infertile donors relative to hAD-MScs without any treatment (control group). RA treatment (hAD-MScs treated with 20 ng/ml BMP4 then treated with 10 ${}^{-6}$ M RA), RA/SCCM treatment (hAD-MScs treated with 20 ng/ml BMP4 then treated with a combination of 10 ${}^{-6}$ M RA and SCCM), SCCM treatment (hAD-MScs treated with SCCM alone).

Interestingly, treatment of hAD-MScs with RA, RA/SCCM, and SCCM led to a decrease in *OCT4* expression at day 19 compared to the control group (Figure 10A), a significant decrease was shown in RA/SCCM and SCCM groups (p = 0.01). While at day 26, the *OCT4* expression was significantly higher in the RA/SCCM and SCCM groups compared to control (p = 0.01) (Figure 10B). However, no significant change was observed in *OCT4* expression between 19 and 26 days of treatment (p = 0.30).

The *SCP3 *was shown to be a mitotic marker whose expression is essential for the development of the synaptonemal complex in homologous chromosomes and was detected following treatment of hAD-MScs with RA, RA/SCCM, and SCCM after 19 days of treatment when compared to the untreated hAD-MScs. On day 19, the group receiving RA treatment had significantly higher levels of *SCP3* expression than the other groups (p = 0.03). However, the difference was not statistically significant in RA/SCCM and SCCM groups (p = 0.93) (Figures 10C and D).

The expression of *SCP3* was found to be significantly higher on day 26 in the SCCM group compared to other groups in fertile donor treated cells (p = 0.02) (Figure 10C), while in infertile donor treated cells, the *SCP3* expression was higher in RA treated group (Figure 10D). Although the overall expression of *SCP3* was not significantly different between 19 and 26 days of treatment (p = 0.14).

Similarly, the post-meiotic spermatid marker *PRM1* (which indicates chromosome condensation) was evidently expressed after SCCM induction at day 19 of culture (p = 0.02), as shown in (Figure 10E-F), its slight expression was also observed in the RA group on infertile donor treated AD-MSCs 19 days after culture (Figure 10F). On day 26 of treatment, *PRM1* expression was detected in treatment and control groups; however, compared to other groups, the SCCM-treated group had a high expression level (p = 0.04) (Figure 10F). Total expression differences of *PRM1 *between 19- and 26-days treatment were not statistically significant (p = 0.06). The expression of the pre-meiotic marker, *STRA8* was not detected neither in treated nor in untreated groups after 19 and 26 days of treatment (Figures 9A and B).

## 4. Discussion 

Stem cells are deemed as prospective innovative therapeutic mechanisms for treating infertility because of their limitless sources and high differentiation capacity (13). Therefore, in this research, we aimed to assess and compare the ability of hAD-MScs to differentiate into germ-line cells in a medium including rabbit SCs conditioned media either with or without additional factors such as RA and BMP-4. The age of the hAD-MScs donors was adopted under 40 as it was reported that increased donor age can adversely affect the growth rate and differentiation capacity of hAD-MScs (14). All the morphological and cell surface markers characteristics of the isolated hAD-MScs are consistent with those of MSCs reported in earlier studies performed on hAD-MScs by other researchers, in addition they were characterized by their capacity to attach to the bottom of the culture flask (15, 16).

Different combinations were used to transdifferentiate hAD-MScs into germ-like cellsin vitro, RA with BMP4 pretreatment, RA/SCCM with BMP4 pretreatment, and SCCM and was evidenced by the detection of expression of the GC markers, *OCT4*, *STRA8*, *SCP3,* and *PRM1*. Herein, to achieve the most effective BMP4 and RA concentrations for the purpose of GC differentiation in MSCs, we examined 5 concentrations of BMP4 and 4 concentrations of RA and revealed that 20 ng/ml BMP4 and 1 µM RA yielded the highest efficiency. According to several studies, effective in vitro development of hAD-MScs into germ-like cells requires the elevation of expression of particular genes linked to GCs differentiation (17, 18).

*OCT4 *is a member of the family of POU transcription factors, is critical for controlling pluripotency throughout embryogenesis and present in both early GCs and pluripotent stem cells (19). *OCT4* has been identified as a pluripotency marker, and its expression might be utilized to assess GC differentiation (16). The expression of *OCT4* was decreased after 19 days of treatment in our study. However, after 26 days of treatment, an increase in its expression was found in RA/SCCM and SCCM treatment groups. This reduction of expression at day 19, and the increase of expression after 26 days, can be an indication of differentiation into GCs in AD-MSCs which may indicate the presence of early GCs in the treatment groups after 26 days as the *OCT4* is highly expressed in early GCs. A previous study reported that *OCT4* expression was significantly increased after 21 days of induction in comparison to day zero (16).

*SCP3* is a late meiosis-specific molecular construction that serves as a molecular framework for chromosomal synapsis regulation (20). As our results showed, all treated cells expressed *SCP3 *markerat days 19 and 26 of culture, which indicates the presence of GCs in the meiotic stage among the cultured cells. *PRM1* is a post-meiotic gene belonging to the protamine genes family. It participates in numerous critical processes, such as spermatogenesis and sperm maturation, DNA packaging, and chromosome condensation (21). At the time of nuclear condensation, *PRM1* is expressed when histones are replaced by protamine(22). In the current work, prominent expression of this gene was verified, indicating the development of post-meiotic GCs. *PRM1* was detected earlier in hAD-MScs cultured in SCCM than in those cultured in RA or in RA combined with SCCM, suggesting that the initiation of GC differentiation in SCCM was faster than that in the other treatment groups. This indicates that factors and metabolites secreted by SCs induce the hAD-MScs to move toward GC differentiation phases.

These results implicate that rabbit testicular cell secretions could efficiently induce in vitro production of male germ-like cells when hAD-MScs is cultured. Furthermore, at day 26 of induction, rabbit SCCM and RA/SCCM combination treatment increased the expression of MGC-related genes, *SCP3* and *PRM1*. This finding is in line with the result of Dissanayake and his colleagues (23), in which they have reported that *SCP3* and *PRM1* were upregulated during the induction of human WJ-MSCs by RA and SCCM and MSCs were successfully transdifferentiated into post-meiotic GCs using a 2-step induction protocol. Unexpectedly, we observed a little expression for the GC markers (*SCP3* and *PRM1*) in the control groups in which no differentiating factors were used. This may suggest that MSCs may undergo spontaneous differentiation into germ lineage during long-time culture in the basic medium, in literature it was reported that the presence of MSCs in a confluency status could induce the spontaneous differentiation (24).

*STRA8*, an *RA* target gene, has been identified as a GC marker that is expressed mostly during the pre-meiotic stage of developing MGCs, its mRNA levels are reported to be low or absent in post-meiotic cells (25). Also it has been reported that *STRA8 *was only observed during the transition of spermatogonia to spermatocytes (26). Ghorbanlou, with co-workers have showed that *STRA8* expression was detected after 7 days of treatment of BM-MScs with RA and testicular cells (27). In addition, Dissanayake and co-authors have mentioned that slight *STRA8* expression was observed after 2 wk of induction (23), while in our study we evaluate the expression after approximately 3-4 wk, and we did not detect any expression of *STRA8.*

In the current work, we did not recognize any expression of *STRA8*, whereas the expression of meiotic *SCP3* and post-meiotic *PRM1* markers were detected after 19 and 26 days of induction; therefore, we can speculate that the cells in the culture represent a mixed population of meiotic and post-meiotic cells. These findings may indicate the presence of germ-like cells in the culture and would be an encouraging step toward obtaining GCs from an easily accessible tissue.

## 5. Conclusion

In conclusion, this work shows that hAD-MScs differentiated into germ-like cells that exhibited the ability to express GC specific markers under in vitro conditions. Upregulation of MGC markers in induction of hAD-MScs suggests that rabbit SCs possess the potential for differentiating hAD-MScs into germ-like cells. Moreover, culturing hAD-MScs in a medium containing rabbit SCCM results in the earlier and higher expression of MGC specific genes, while the use of RA with rabbit SCCM had no positive effect on the GC specific genes expression.

Altogether, the differentiation potential of hAD-MScs using rabbit testicular cells might be a highly useful tool in the field of regenerative medicine. Additional research into the differentiation of hAD-MScs into MGCs using rabbit SCs might open up a novel and prospective therapy option for reproductive problems, perhaps assisting in the resolution of certain infertility issues.

##  Conflict of Interest

The authors declare that there is no conflict of interest.
